# pH‐sensitive soluble soybean polysaccharide/SiO_2_ incorporated with curcumin for intelligent packaging applications

**DOI:** 10.1002/fsn3.2187

**Published:** 2021-02-25

**Authors:** Davoud Salarbashi, Mohsen Tafaghodi, Bibi Sedigheh Fazly Bazzaz, Seyyed Mohammad Aboutorabzade, Morteza Fathi

**Affiliations:** ^1^ Nanomedicine Research Center School of Medicine Gonabad University of Medical Sciences Gonabad Iran; ^2^ Department of Food Science and Nutrition School of Medicine Gonabad University of Medical Sciences Gonabad Iran; ^3^ Nanotechnology Research Center Pharmaceutical Technology Institute Mashhad University of Medical Sciences Mashhad Iran; ^4^ Pharmaceutics Department School of Pharmacy Mashhad University of Medical Sciences Mashhad Iran; ^5^ Biotechnology Research Center Pharmaceutical Technology Institute Mashhad University of Medical Sciences Mashhad Iran; ^6^ Pharmaceutical Control Department School of Pharmacy Mashhad University of Medical Sciences Mashhad Iran; ^7^ Department of Medicinal Chemistry School of Pharmacy Mashhad University of Medical Sciences Mashhad Iran; ^8^ Health Research Center Life Style Institute Baqiyatallah University of Medical Sciences Tehran Iran

**Keywords:** biodegradable films, intelligent packaging, SiO_2_ nanoparticles, soluble soybean polysaccharide

## Abstract

In the present work, the effect of various concentrations of SiO_2_ nanoparticles (5, 10, and 15%) on physicochemical and antimicrobial properties of soluble soybean polysaccharide (SSPS)‐based film was investigated. Then, the migration of SiO_2_ nanoparticles to ethanol as a food simulant was evaluated. Subsequently, curcumin was added to the nanocomposite formulation to sense the pH changes. Finally, the cytotoxicity of the developed packaging system was investigated. With increasing nanoparticle concentration, the film thickness, water solubility, and water vapor permeability decreased and mechanical performance of the films improved. SSPS/SiO_2_ nanocomposite did not show antibacterial activity. *SEM* analysis showed that SiO_2_ nanoparticles are uniformly distributed in the SSPS matrix; however, some outstanding spots can be observed in the matrix. A very homogeneous surface was observed for neat SSPS film with *R*_a_ and *R*_q_ values of 3.48 and 4.26, respectively. With the incorporation of SiO_2_ (15%) into SSPS film, *R*_a_ and *R*_q_ values increased to 5.67 and 5.98, respectively. Small amount of SiO_2_ nanoparticles was released in food simulant. The nanocomposite incorporated with curcumin showed good physical properties and antibacterial activity. A strong positive correlation was observed between TVBN content of shrimp and a* values of the films during storage time (Pearson's correlation = 0.985).

## Graphical abstract

Neither cytotoxicity nor antimicrobial activity was observed for the SSPS/SiO_2_ nanocomposites. Only a small amount of nanoparticles could release into a food simulant. Incorporation of SiO_2_ nanoparticles improved the physicochemical properties of SSPS*‐based films. Incorporation of SiO_2_ nanoparticles imparts antimicrobial activity of SSPS*‐based films *Soluble soybean polysaccharide (SSPS).

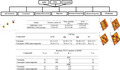

## 1 INTRODUCTION

The use of eco‐friendly materials for food packaging is important in both reducing environmental problems and improving the integrity of foods (Othman, 2014). Therefore, the use of biodegradable materials such as proteins and polysaccharides for the development of eco‐friendly packaging systems has attracted the attention of researchers (Galus & Lenart, 2013; Zolfi et al., 2014). Polysaccharides are inexpensive, safe, renewable, and biodegradable polymers with relatively good physical properties (Akbariazam et al., 2016; Hosseini et al., 2015; Qazanfarzadeh & Kadivar, 2016). However, they have some disadvantages, such as high water solubility and weak mechanical characteristics (Bourtoom, 2009; Hassan et al., 2018; Tajik et al., 2013). Various techniques have been used to overcome these weaknesses. Incorporating nanoparticles such as SiO_2_ into the films is one of the most common techniques that improve the film's mechanical strength (Givi et al., 2010). Furthermore, the use of nanoparticles in film formulation can preserve foods against microbial spoilage with their antimicrobial and antifungal effects during storage period (Ghazihoseini et al., 2015; Kanmani & Rhim, 2014; Oliveira et al., 2016; Qazanfarzadeh & Kadivar, 2016; Zolfi et al., 2014).

Curcumin is a polyphenolic compound derived from the ground rhizomes of *Curcuma longa* L. Curcumin possesses diverse biological effects such as antimicrobial, antioxidant, and anti‐HIV activities (Solghi, Emam‐Djomeh, Fathi, & Farahani, 2020). It also has been reported that this bioactive compound can be used in treating Parkinson's and Alzheimer's patients (Robles‐Almazan et al., 2017). The bioactive compounds of curcumin into eco‐friendly packaging have attracted the attention of scientists in recent years (Jafarzadeh et al., 2020; Mei et al., 2020). In recent years, various works have been focused on developing new intelligent packaging systems based on biopolymers. Mei et al. (2020) developed a pH‐sensitive film based on sago starch incorporated with anthocyanin‐rich torch ginger extract. They observed that the color of films altered from pink to green when the pH increased from pH 4 to 9. In another study, Arezoo et al. (2020) evaluated the synergistic effects of *cinnamon* essential oil and TiO_2_ nanoparticles on antimicrobial and functional characteristics of sago starch films.

Soluble soybean polysaccharide (SSPS) is a water‐soluble polysaccharide that is extracted from cell wall material of the cotyledon of soybeans. It has been reported that this polysaccharide can be used as a thickener, stabilizer, and emulsifying agent (Maeda & Nakamura, 2009). To date, various studies have been focused on the fabrication of SSPS‐based films (Alipoormazandarani et al., 2015; Shaili et al., 2015; Tajik et al., 2013). However, there are no studies yet leading to the manufacture of smart packaging systems that are SSPS/SiO_2_/curcumin‐based.

The present study aimed to fabricate SSPS‐based films incorporated with different concentrations of SiO_2_ nanoparticles and subsequently investigate the cytotoxicity and physicochemical and biological characteristics of the films. The antibacterial and antimold activities of the films were also analyzed. Finally, the nanocomposite fabricated at optimal condition was incorporated by curcumin to sense pH changes.

## 2 MATERIALS AND METHODS

### 2.1 Materials

Soluble soybean polysaccharide (SSPS) was obtained from Fuji Oil Company (Osaka, Japan). All the chemicals used in the present research were purchased from Sigma‐Aldrich. SiO_2_ nanoparticles with size of 20–50 nm were obtained from Rockwood Additives Ltd. Microbial cultures were obtained from Himedia. Double distilled water was used.

### 2.2 Methods

#### 2.2.1 Fabrication of SSPS/SiO_2_ nanocomposites

Preparation of SSPS/SiO_2_ nanocomposites was carried out as follows:

1. To prepare nanoparticle suspensions, SiO_2_ with different concentrations was dispersed in hot water (80°C) under magnetic stirring for 2 hr.
2. The SSPS (2.4 g) was dispersed in distilled water (40 ml) under mechanical stirring (300 rpm, at 25°C for 4 hr), followed by storage at 4°C for overnight.
3. The glycerol (35 wt%) as plasticizer was incorporated into polymer solution under magnetic stirring and then sonicated for 10 min.
4. The prepared nanoparticle suspensions were incorporated into the resulting solution and homogenized for 15 min.
5. The resulting dispersions were cast on petri dishes, dried at 25°C for 24 hr, and kept at zip kips for further evaluation.

#### 2.2.2 Preparation of smart nanocomposite based on SSPS/SiO_2_/curcumin

SSPS powder (2.4 g) was dispersed in distilled water (40 ml) at 25°C and then kept at 4°C overnight to be hydrated completely. Curcumin (5 mg) was dissolved in 2 ml ethanol (95%) containing Tween 80 under vortexing for 3 min, followed by solvent evaporation with rotary evaporator. The curcumin solution was added to the SSPS dispersion with flow rate of 1 ml/min and vortexed for 5 min. Finally, the resulting solution was cast on petri dishes, dried at 25°C for 20 hr, peeled, and stored at zip kips until further evaluations (Taghinia, Abdolshahi, Sedaghati, & Shokrollahi, 2021).

#### 2.2.3 Physical characteristics

##### Film thickness

The thickness of the films was determined using a handheld digital micrometer. For this purpose, 10 random points were selected for the measurements and the average values were reported.

##### Water solubility (WS), moisture content (MC), and water vapor permeability (WVP)

The films were cut into 2.0 × 2.0 cm^2^ pieces and dried at 105°C using an oven‐drier until they reached a constant weight. The dried samples were weighed to determine the initial dry weight (W0). Afterward, the dried samples were immersed in 50 ml distilled water at 25°C for 6 hr and the remaining pieces were taken out and dried (at 105°C) until they reached to a constant weight (W1). WS was computed as follows:

(1) $$\text{WS}\left(\%\right)=\frac{W_{0}‐W_{1}}{W_{0}}$$

To determine moisture content (MC) of the films, they were heated at 105°C for 24 hr to reach a constant weight. MC values were calculated using the following formula (Zolfi et al., 2014).

(2) $$\text{MC}\left(\%\right)=\frac{\text{weight of water}}{\text{total weight of sample}}\times 100$$

WVP was determined following ASTM E96/E96M‐16 standard test (ASTM, 2004). Briefly, the films were cut and mounted horizontally on cups containing calcium anhydride. The cups were placed in a desiccator containing saturated sodium chloride solution (NaCl—75% RH) and weighed periodically for 4 days. WVP of the samples was calculated as follows (Eq. 2):

(3) $$\text{WVP}=\frac{\Delta m}{A\Delta t}\frac{X}{\Delta p}$$

where Δ*m*/Δ*t*, *X*, and *A* are the weight of moisture gain per unit of time (g/s), the film thickness (mm), and the film surface area (m^2^).

#### 2.2.4 Mechanical properties

The mechanical characteristics of the films were analyzed by an M350‐10CT Machine (Testometric Co., Ltd.) at a cross‐head speed of 10 mm/min at 25°C. The test was undertaken as described by ASTM D^882^‐^91^. The measurements were done four times, and the average values were reported. Tensile strength (TS) and elongation at break (EB) were determined using the following equations:

(3) $$\text{TS}=\frac{F_{\max}}{A_{\min}}$$

(4) $$\text{EB}\left(\%\right)=\frac{L_{\max}}{L_{0}}\times 100$$

where *F*_max_, *A*_min_, *L*_max_, and L0 represent the maximum load, the cross‐sectional area, the extension at rupture point, and the original length of the specimen, respectively.

#### 2.2.5 Scanning electron microcopy (*SEM*) analysis

*SEM* analysis was carried out on the surfaces and cross sections of the specimens. Before the analysis, the nanocomposite surface was coated by gold layer with a sputter coater. The analysis was conducted using an accelerating voltage of 20.0 kV.

#### 2.2.6 Atomic force microscopy (AFM)

AFM was employed to investigate the topological properties of neat SSPS film and the film incorporated by different concentrations of SiO_2_ nanoparticles. The surface parameters of the films including root‐mean‐square roughness (Rq) and average roughness (Ra) were determined according to ASME B46.1 2009 Standard (1995).

#### 2.2.7 Evaluation of the nanoparticle migration

The released SiO_2_ nanoparticles into ethanol were evaluated after dipping nanocomposite films in simulant solution. The Si content in the applied food simulant was determined using ASTM D 4754‐11 method. The concentration of Si in ethanol was measured using inductively coupled plasma optical emission spectrometry (ICP‐OES) (Salarbashi et al., 2018a).

#### 2.2.8 Antibacterial activity

The antibacterial activities of neat SSPS, SiO_2_, SSPS/SiO_2,_ and SSPS/SiO_2_/curcumin nanocomposites were studied as their inhibitory and bactericidal effects against the growth of *Staphylococcus aureus* PTCC 1,112 (ATCC 6538p), *Bacillus subtilis* PTCC 1,023 *(*ATCC 6,633), and *Staphylococcus epidermidis* PTCC 1,114 (ATCC 12,228) and DSMZ 3,270. SSPS solution with distinct concentration (16 mg/ml) and predetermined concentration of SiO_2_ (preliminary experiments with different concentrations of SiO_2_ did not show any antibacterial effects), SSPS/SiO_2,_ and SSPS/SiO_2_/curcumin nanocomposites, and various concentration of curcumin were incorporated into sterilized Mueller Hinton Broth (MHB) and then incubated to perform in vitro antibacterial screening. Culture media and media plus bacterial strain were employed as negative and positive controls, respectively. Antimicrobial parameters of the tested samples including minimal inhibitory concentration (MIC) and minimal bactericidal concentration (MBC) were measured as earlier reported protocol (Salarbashi et al., 2018).

#### 2.2.9 The response of smart film to pH changes

Digital camera (Kodak M853, USA) was employed to observe the photographs of smart films after 30‐min contacting with media with various pHs. Color changes were monitored using a colorimeter. ImageJ software was used to determine color properties of the films (Abedinia et al., 2018).

#### 2.2.10 Cytotoxicity

In order to properly investigate the cellular toxicity of SiO_2_ nanoparticles, curcumin, and curcumin plus SiO_2_ nanoparticles, alamarBlue test, which is based on colorimetric and "oxidation and reduction" reaction, was applied on different cell lines. The substance was adjacent to the cells for 72 hr at 37°C incubator, which was saturated with humidity and 5% CO_2_. The absorbance was then read by a microplate spectrophotometer reader at 570 and 600 nm.

#### 2.2.11 Application of the films as an indicator for freshness monitoring of shrimp

For this purpose, SSPS/SiO_2_/curcumin films were cut and then placed on 6.0 ± 0.3 g shrimps kept in petri dishes. The samples were stored in an incubator at 25°C and 75% RH. The total volatile basic nitrogen (TVBN) content of the shrimps was determined during storage period by stream distillation method as described by Cai et al. (2011).

#### 2.2.12 Statistics

The experimental results were analyzed using complete randomized design using SPSS software (Version 16; SPSS Inc., Chicago, USA). In order to compare the mean values, Duncan's test was used (*α* = 0.05).

## 3 RESULTS AND DISCUSSION

### 3.1 Physicomechanical, morphological, and antibacterial properties of SSPS‐based films incorporated by SiO_2_

#### 3.1.1 Physical properties

The effect of SiO_2_ nanoparticle concentration on physical properties of the developed nanocomposites is given in Table 1. An increase in the concentration of SiO_2_ nanoparticle was accompanied by a decrease in the film thickness. This effect is attributed to the increasing compactness of polysaccharide‐based film structure at higher nanoparticle level. This compactness can change the physicomechanical properties of films. As presented in Table 1, the incorporation of SiO_2_ nanoparticles into SSPS film led to decrease in the moisture content and water solubility. This observation has been associated with the formation of H‐bonding between the hydroxyl groups of polysaccharide with SiO_2_ nanoparticles. These interactions reduce the availability of hydroxyl groups of polysaccharide interacting with water molecules and as a result decrease the water solubility of the films (Shaili et al., 2015). Furthermore, this effect may be related to more compact structure of the films with higher nanoparticle content.

**TABLE 1:** Physical characteristics of the developed SSPS films as a function of SiO_2_ nanoparticle concentration*

SiO_2_ content (%)	Thickness (mm)	Moisture content (%)	Water solubility (%)	WVP (×10^6^ gm^−1^ s^−1^ pa^−1^)
0	0.201 ± 0.003^a^	21.02 ± 0.04^a^	70.07 ± 0.07^a^	7.06 ± 0.249^b^
5	0.180 ± 0.001^b^	16.12 ± 0.11^b^	68.64 ± 0.62^b^	5.93 ± 0.005^c^
10	0.161 ± 0.004^c^	15.91 ± 0.02^c^	65.90 ± 0.08^c^	7.46 ± 0.017^b^
15	0.142 ± 0.005^d^	15.86 ± 0.06^c^	65.45 ± 0.04^c^	8.97 ± 0.004^a^

* a, b, c, and d: Different letters in the same column indicate significant differences at 5%.

**FIGURE 1:**
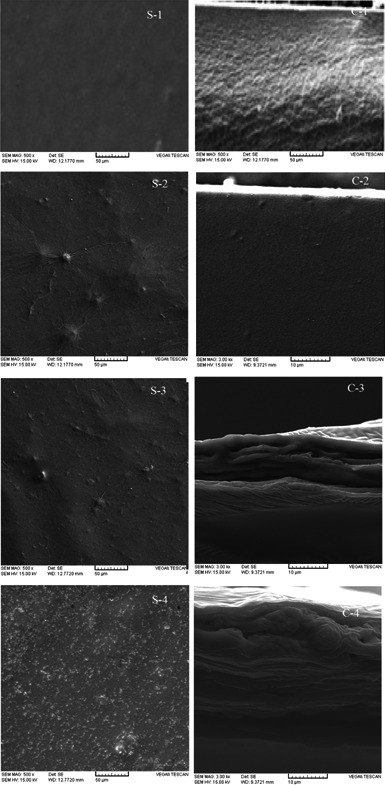
The *SEM* images of SiO_2_‐free SSPS (S1 and C1), SSPS/SiO_2_ 1% (S2 and C2), SSPS/SiO_2_ 3% (S3 and C3), and SSPS/SiO_2_ 5% (S4 and C4). The letters of C and S stand for “cross‐section” and “surface” profiles of the composites, respectively

Water vapor permeability (WVP) is a key factor that must be considered when designing food packaging systems (Abdollahi et al., 2013). When the SiO_2_ concentration increased up to 5%, the WVP decreased, but with further increase in the concentration of SiO_2_, WVP increased. The addition of nanoparticles to polymeric matrix prolongs the diffusive pathways of water vapor and thus leads to decrease in water vapor permeability (Nielsen, 1967). The increase in WVP at higher nanoparticle concentration could be due to nonuniform distribution of nanoparticles and formation of aggregated complex, which was confirmed by *SEM* analysis (Figure 1) (Abdollahi et al., 2013).

Physicomechanical properties of packaging systems are strongly dependent on their morphology and microstructure (Fabra et al., 2011). The surface and cross‐sectional images of pure films and those incorporated by SiO_2_ nanoparticles are shown in Figure 1. From *SEM* images, it is clear that SiO_2_ nanoparticles are uniformly distributed in the SSPS matrix; however, some outstanding spots can be observed in the matrix. This observation is due to the agglomeration of SiO_2_ nanoparticles at high nanoparticle concentration. The aggregation of nanoparticles in hydrophilic matrix is due to their high surface energy that facilitates the coagulation process and the strong cohesive forces between their primary particles. The similar results have been reported in our previous study that showed SSPS had poor affinity with ZnO nanoparticles (Salarbashi et al., 2016). As observed in Figure 1, the entire surface of the films incorporating 15% SiO_2_ covered by nanoparticles can act as barrier for diffusion of gasses. Furthermore, it can improve the mechanical properties of the films.

**FIGURE 2:**
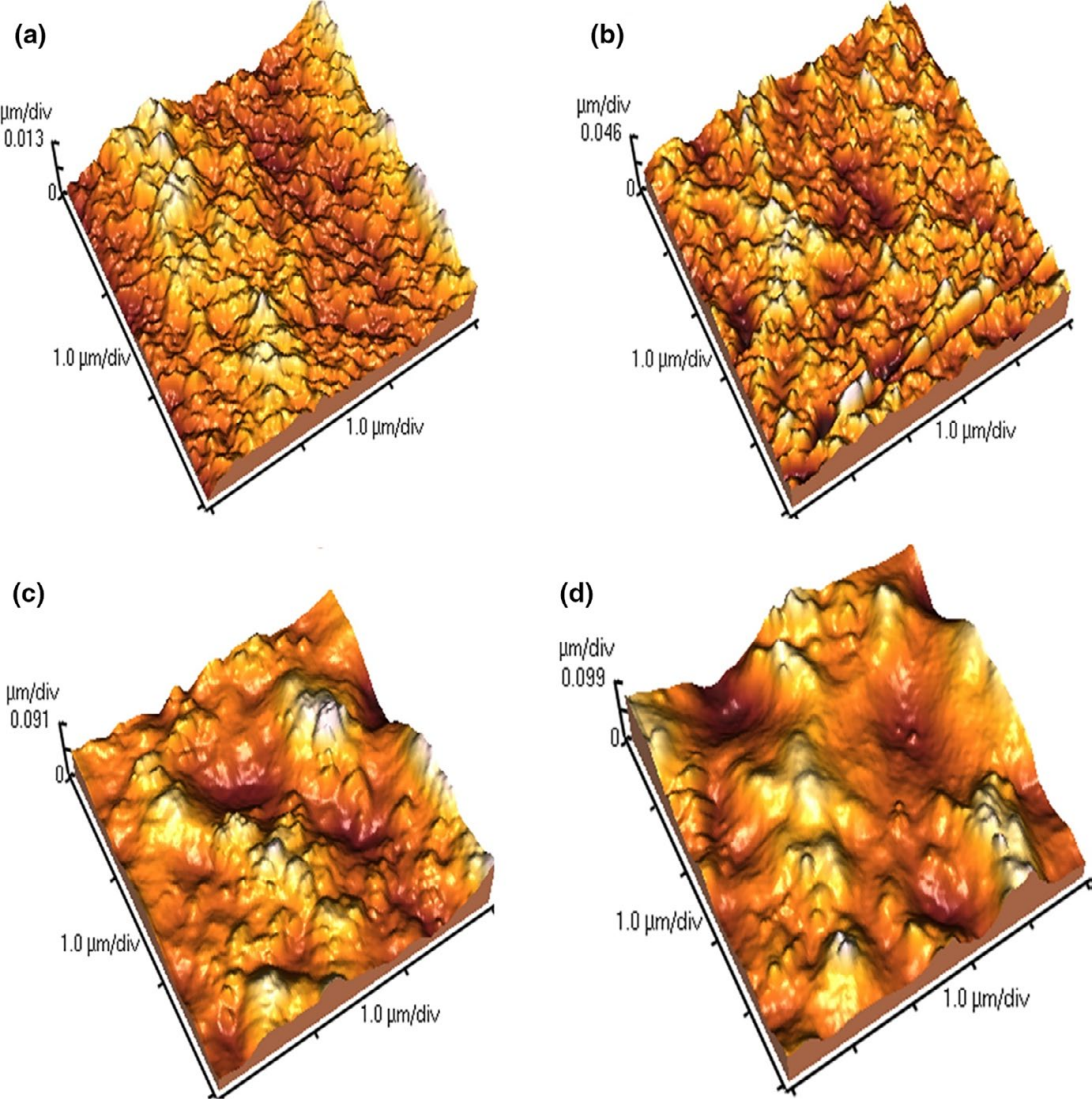
The AFM micrographs of SiO_2_‐free SSPS (a), SSPS/SiO_2_ (1%) (b), SSPS/SiO_2_ (3%) (c), and SSPS/SiO_2_ (5%) (d)

In the present study, AFM was employed to evaluate the topological properties of the developed films. The AFM images of neat SSPS and SSPS/SiO_2_ films incorporated with various concentrations of SiO_2_ are presented in Figure 2. A very homogeneous surface was observed for neat SSPS film with *R*_a_ and *R*_q_ values of 3.48 and 4.26, respectively. With incorporation of SiO_2_ into SSPS film, a considerable increase was observed in the surface roughness of the film specimens. This observation is consistent with that observed in *SEM* analysis described above. Topological characteristics have remarkable effect on the appearance of films. For instance, the films with more surface roughness have low degree of transmittance. Furthermore, it has also reported that the films with more surface roughness may possibly have an open and porous structure, which results in lower hardness. But, we observed that the mechanical properties of the films increased as the concentration of SiO_2_ nanoparticles increased. The reason for that may be due to the covering of entire surface of the films incorporated by SiO_2_ nanoparticles that reinforce the mechanical properties of the films.

#### 3.1.2 Mechanical characteristics

Since mechanical properties have considerable effect on consumer acceptance and preservation of food product, SiO_2_ nanoparticles were incorporated into the SSPS films to reinforce the mechanical properties of the films. The mechanical characteristics of SSPS films incorporated with different concentrations of SiO_2_ nanoparticles are presented in Table 2. According to the available literature, nanoparticles can strongly interact with functional groups of polymers and give robust characteristics to the biopolymer films. As shown in Table 2, with the addition of SiO_2_ nanoparticles, tensile strength (maximum resistance of film against the applied stress) and elongation at break of the SSPS films increased significantly. The improved TS of the films incorporated with SiO_2_ nanoparticles may be related to the strong interaction between the nanoparticles and SSPS structure by covalent bond (Jia, Li, Cheng, Zhang, & Zhang, 2007). Increasing EB with increase in SiO_2_ nanoparticles has been attributed to the plasticizing role SiO_2_ nanoparticles. SiO_2_ nanoparticle homogeneously dispersed in the molecular skeleton of matrix, which might influence intra‐ or interaction of polysaccharide chain. This was conducive to the mutual rearrangements of chain unit, thereby improving flexibility of molecular chains (Hou et al., 2019).

**TABLE 2:** Mechanical characteristics of SSPS films incorporated with various concentrations of SiO_2_ nanoparticles*

SiO_2_ content (wt. %)	Tensile strength (MPa)	Elongation at break (% )
0	12.71 ± 0.02^d^	30.15 ± 0.02^b^
5	15.74 ± 0.03^c^	25.31 ± 0.03^c^
10	17.05 ± 0.06^b^	31.85 ± 0.06^b^
15	19.70 ± 0.01^a^	38.32 ± 0.01^a^

* a, b, c, and d: Different letters in the same column indicate significant differences at 5%.

#### 3.1.3 Antibacterial activity of SSPS, SiO_2_, and SSPS/SiO_2_ nanocomposites

SSPS (16 mg/ml) and different concentrations of SiO_2_ (0%–15% w/w) and SSPS/SiO_2_ nanocomposites without curcumin did not show clear antibacterial activity against *Staphylococcus aureus* PTCC 1,112 (ATCC 6538p), *Bacillus subtilis* PTCC 1,023 *(*ATCC 6,633), *Staphylococcus epidermidis* (DSMZ 3,270), and *Staphylococcus epidermidis* PTCC 1,114 (12,228) (DSMZ 3,270) (data not shown).

#### 3.1.4 Cytotoxicity

MTT test indicated no profound cytotoxicity on the cancerous cells evaluated (A549, cho, hek, and 4t1). The result demonstrated that the concentration of SiO_2_ nanoparticles that could show cytotoxicity was far greater than those employed in the tested nanocomposites. Therefore, these nanocomposites do not have potential of carcinogenicity. This result is in agreement with the result reported by Salarbashi et al. (2018) who demonstrated that SSPS/TiO_2_ nanoparticles had no cytotoxicity.

#### 3.1.5 Release of SiO_2_ nanoparticles into food simulant

The release of SiO_2_ nanoparticles into the 10% ethanol was evaluated to determine the safety of fabricated nanocomposites. The concentration of Si in the solvent containing pure SSPS was below the method's detection limit (MDL). On the other hand, the final content of Si in 10% ethanol containing SSPS/SiO_2_ (15%) was found to be 7.08 ± 0.03 ppm, demonstrating the migration of nanoparticles from the developed nanocomposite. It has been reported that the small amount of Si released from nanocomposite may be due to the strong interaction between SiO_2_ nanoparticles and SSPS (Salarbashi et al., 2018b). The similar observations have been reported as the main reason for small amount of nanoparticles released from nanocomposites (Lin et al., 2014; Salarbashi et al., 2018b).

### 3.2 Physical properties, antimicrobial activity, and cytotoxicity of SSPS/SiO_2_/curcumin

Based on the previous studies, curcumin has strong antimicrobial and antioxidant activities. Furthermore, the films loaded by curcumin are sensitive to pH changes and thus can be used to sense the food quality. Therefore, in the present study, first curcumin was incorporated into the film constructed at optimal concentration of SiO_2_ (15%), and then, physical and antimicrobial properties and cytotoxicity of the developed films were examined. Finally, the color change in the developed films exposing liquid with different pHs was tested.

#### 3.2.1 Physical properties

Several attempts have been conducted on the improvement of polysaccharide‐based films. The use of hydrophobic compounds in film formulation is one of the most effective approaches to decrease water solubility. The effect of curcumin addition on physical properties of SSPS/SiO_2_ (15%) and SSPS/SiO_2_ (15%) nanocomposite films is given in Table 3. As expected, the water solubility, moisture content, and water vapor permeability of the nanocomposite decreased when the curcumin was incorporated into film formulation. This effect is related to the hydrophobic nature of curcumin. The interaction between curcumin and polysaccharides leads to the improvement of water barrier properties of the films. Furthermore, curcumin crystals act as a hindrance for water molecules and decrease WVP (Liu et al., 2016). Similar observations have been reported for the effect of curcumin on physical properties of the films produced by gelatin (Liu et al., 2016), k‐carrageenan (Liu et al., 2018), and *Melissa officinalis* seed gum (Rostami & Esfahani, 2019). Comparatively, the value of WVP of SSPS/SiO_2_ (5%)/curcumin nanocomposite films (0.14 × 10^−6^ gm^−1^ s^−1^ pa^−1^) was higher than those reported for synthetic‐based films such as cellophane (8.4 10^–1^1 g/m s^–1^ Pa^–1^), LDPE (3.6 10^–13^ g/m s^–1^ Pa^–1^), and PVDC (2.2 10^–13^ g/m s^–1^ Pa^–1^) [25]. But, the WVP of SSPS/SiO_2_ (15%)/curcumin nanocomposite films was lower than most of the biopolymer‐based films. Accordingly, the developed films can be suggested as appropriate candidate for application in packaging industry.

**TABLE 3:** Physical properties of SSPS/SiO_2_ (15%)/curcumin films

Sample	Moisture content (%)	Water solubility (%)	WVP (×10^6^ gm^−1^ s^−1^ pa^−1^)
SSPS/SiO_2_ (15%)	15.86 ± 0.06^a^	65.45 ± 0.04^a^	8.97 ± 0.00^a^
SSPS/SiO_2_ (15%)/curcumin	7.01 ± 0.52^b^	38.17 ± 1.98^b^	0.14 ± 0.21^b^

a, b, c, and d: Different letters in the same column indicate significant differences at 5%.

#### 3.2.2 Antibacterial activity

Antibacterial activity of curcumin has been well documented in the literature (Liu et al., 2016; Varaprasad et al., 2011; Wu et al., 2019). Antimicrobial activity of films containing curcumin against several strains of bacteria was evaluated. The values of minimum inhibition concentration (MIC) (the lowest concentration of antimicrobial agent that prevents visible growth of bacterium) and minimum bactericidal concentration (the lowest concentration that an antimicrobial agent will kill a microorganism) of curcumin and SSPS/SiO_2_ (15%)/curcumin nanocomposites are shown in Table 4. It was observed that the developed film loaded by curcumin effectively suppressed the growth of tested bacteria. Phenolic compounds such as curcumin interact with membrane proteins of bacterial cells and as a result inhibit bacterial growth (Haslam et al., 1988). Likewise, Varaprasad et al. (2011) showed that curcumin‐loaded CMC films could inhibit the growth of *E. coli*. In another study, Liu et al. (2016) demonstrated the chitosan‐based film incorporated with curcumin had antibacterial and antimold activity. From Table 4, it is evident that the antimicrobial activity of curcumin‐loaded nanocomposite was more than pure curcumin, indicating a synergistic effect between curcumin and SiO_2_ nanoparticles. In conclusion, SSPS/SiO_2_ (15%)/curcumin nanocomposite films can be used as packaging system to delay or prevent the bacterial growth.

**TABLE 4:** The values of MIC and MBC of curcumin and SSPS/SiO_2_ (15%)/curcumin nanocomposites

Compounds	Bacteria: PTCC number or DSMZ			
	S. e.1	S. e.2	B. s.	S. a.
	MIC			
Curcumin	31	125	31	31
Curcumin + nanocomposite	62	500	62	62
	MBC			
Curcumin	31	125	125	31
Curcumin + nanocomposite	31	125	125	31

Note

S. a.: *Staphylococcus aureus* (PTCC 1,112), S. e. 1.: *Staphylococcus epidermidis* (PTCC 1,114), S. e.2: *S. epidermidis* (DSMZ 3,270), B. s.: *Bacillus subtilis* (PTCC 1,023), PTCC, Persian Type Culture Collection.

#### 3.2.3 Cytotoxicity

An ideal packaging should not cause any adverse effects, which could be examined through in vitro cytotoxicity tests. The cytotoxicity effects of curcumin and SSPS/SiO_2_ (15%)/curcumin nanocomposites on the cell lines (A549, cho, hek, and 4t1) were evaluated. Curcumin and SSPS/SiO_2_ (15%)/curcumin nanocomposites showed cytotoxicity on the cancerous cells evaluated (A549, cho, hek, and 4t1) at high dose (Table 5).

**TABLE 5:** The cytotoxicity of curcumin and SSPS/SiO_2_ (15%)/curcumin nanocomposites

Compounds	Cell line			
	A549	cho	hek	4t1
Curcumin	61.1 ± 6.5	81.83 ± 7.8	84.68 ± 9.5	62.8 ± 6.5
Curcumin + SSPS nanocomposite	56.45 ± 5.4	70.54 ± 7.4	65.49 ± 12.5	50 ± 7.2

Note

IC_50_ of curcumin and the curcumin‐loaded nanocomposite (μg/ml).

### 3.3 Film response to pH changes

**FIGURE 3:**
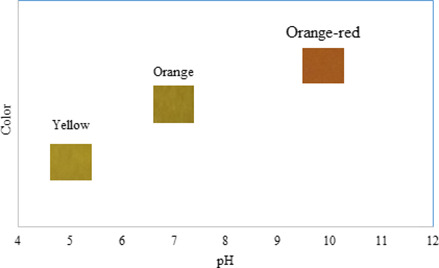
The pH response of the smart film in contact with acid (a), neutral, (b) and alkali (c) conditions

Curcumin is a pH‐sensitive compound that can inform consumer the freshness of food product. For instance, in the animal‐based protein, the bacterial spoilage leads to producing TVBN that increases the medium pH (Wannawisan, Sane, Runglerdkriangkrai, Wilaipun, & Suppakul, 2018). The color change in SSPS/SiO_2_ (15%)/curcumin nanocomposite films exposing acidic, neutral, and alkaline medium is depicted in Figure 2. It is evident that as the pH increased, the color of the films tended to red, revealing the developed smart packaging can be used to detect the pH change occurred as a result of basic spoilage. At alkaline medium, some of the phenolic hydroxyl groups of curcumin deprotonated and thus make the molecule more polar. This leads to change in the color of curcumin (Figure 3).

### 3.4 Shrimp spoilage trial

**FIGURE 4:**
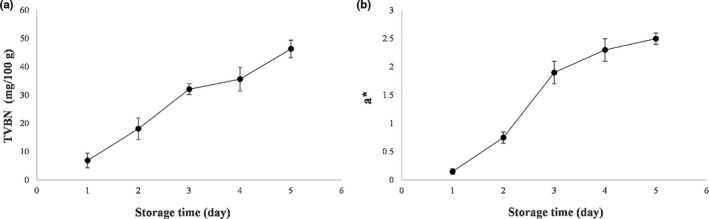
Application of LISG/curcumin film for monitoring shrimp freshness (a: a* values of the films over storage time and b: TVBN content of shrimp samples over storage time)

It has been documented that the major reason for the spoilage of animal‐based protein foods is microorganisms. Total volatile basic nitrogen (TVBN) is produced during spoilage of seafood. TVBN content is known as a chemical indicator for determining the extent of spoilage of seafood. In the present work, the correlation between color changes in SSPS/SiO_2_ (15%)/curcumin film and TVBN content produced over storage time was investigated. As shown in Figure 4a, in the initial, the TVBN content of fresh shrimp was 6.89 mg/100 g and increased to 46.33 mg/100 g when storage time increased from 0 to 5 days. Furthermore, with increasing storage time, a clear color change was observed for the developed films. The value of a* (redness) as a function of storage period is shown in Figure 4. Pearson's correlation test was performed to determine whether there was a significant correlation between the TVBN content and a* values of the films during storage time. A strong positive correlation was observed between TVBN content of shrimp and a* values of the films during storage time (Pearson's correlation = 0.985). Thus, SSPS/SiO_2_/curcumin can be introduced as a promising intelligent packaging system to monitor the shrimp freshness.

## 4 CONCLUSION

The pH colorimetric indicator films based on curcumin/SSPS/SiO_2_ nanoparticles were fabricated for freshness monitoring of shrimp. The water solubility, moisture content, and water vapor permeability of the nanocomposite decreased when the curcumin was incorporated into film formulation. The films incorporated with curcumin had good antibacterial activity that makes it good candidate to apply as antimicrobial packaging system. The developed smart packaging system could successfully sense the changing pH and therefore can be used to detect the pH change occurred as a result of basic spoilage. Curcumin/SSPS/SiO_2_ nanoparticle films exhibited distinctive color change to indicate spoilage of shrimp during storage. In conclusion, SSPS/curcumin/SiO_2_ nanoparticle films with high antibacterial activity and capability to detect the pH change of packaged foods have a high potential for active and intelligent packaging.

## CONFLICT OF INTEREST

We declare that there are no conflicts of interest.

## Data Availability

Data are available on request due to privacy/ethical restrictions.
